# A General Strategy to Endow Natural Fusion-protein-Derived Peptides with Potent Antiviral Activity

**DOI:** 10.1371/journal.pone.0036833

**Published:** 2012-05-16

**Authors:** Antonello Pessi, Annunziata Langella, Elena Capitò, Silvia Ghezzi, Elisa Vicenzi, Guido Poli, Thomas Ketas, Cyrille Mathieu, Riccardo Cortese, Branka Horvat, Anne Moscona, Matteo Porotto

**Affiliations:** 1 PeptiPharma, Pomezia, Rome, Italy; 2 Merck Serono, Guidonia Montecelio, Rome, Italy; 3 BASF Italia, Pontecchio Marconi, Bologna, Italy; 4 Viral Pathogens and Biosafety, Division of Immunology, Transplantation and Infectious Diseases, San Raffaele Scientific Institute, Milan, Italy; 5 AIDS Immunopathogenesis Units, Division of Immunology, Transplantation and Infectious Diseases, San Raffaele Scientific Institute, Milan, Italy; 6 Vita-Salute San Raffaele University, School of Medicine, Milan, Italy; 7 CEINGE, Naples, Italy; 8 INSERM, Ecole Normale Supérieure de Lyon, Lyon, France; 9 IFR128 BioSciences Lyon-Gerland Lyon-Sud, University of Lyon, Lyon, France; 10 Department of Microbiology and Immunology, Weill Medical College of Cornell University, New York, New York, United States of America; 11 Pedriatics, Weill Medical College of Cornell University, New York, New York, United States of America; Lady Davis Institute for Medical Research, Canada

## Abstract

Fusion between the viral and target cell membranes is an obligatory step for the infectivity of all enveloped virus, and blocking this process is a clinically validated therapeutic strategy.

Viral fusion is driven by specialized proteins which, although specific to each virus, act through a common mechanism, the formation of a complex between two heptad repeat (HR) regions. The HR regions are initially separated in an intermediate termed “prehairpin”, which bridges the viral and cell membranes, and then fold onto each other to form a 6-helical bundle (6HB), driving the two membranes to fuse. HR-derived peptides can inhibit viral infectivity by binding to the prehairpin intermediate and preventing its transition to the 6HB.

The antiviral activity of HR-derived peptides differs considerably among enveloped viruses. For weak inhibitors, potency can be increased by peptide engineering strategies, but sequence-specific optimization is time-consuming. In seeking ways to increase potency without changing the native sequence, we previously reported that attachment to the HR peptide of a cholesterol group (”cholesterol-tagging”) dramatically increases its antiviral potency, and simultaneously increases its half-life in vivo. We show here that antiviral potency may be increased by combining cholesterol-tagging with dimerization of the HR-derived sequence, using as examples human parainfluenza virus, Nipah virus, and HIV-1. Together, cholesterol-tagging and dimerization may represent strategies to boost HR peptide potency to levels that in some cases may be compatible with *in vivo* use, possibly contributing to emergency responses to outbreaks of existing or novel viruses.

## Introduction

Fusion between the viral and the target cell membrane is an obligatory step for the infectivity of all enveloped viruses. The development of compounds – peptides in particular – which block this process is a well-established therapeutic strategy [1] that has been clinically validated for the retrovirus human immunodeficiency virus type 1 (HIV-1) with the development of the peptide fusion inhibitor enfuvirtide (Fuzeon®, also known as T20) [2].

Viral fusion is driven by specialized proteins which, although specific to each virus, act through a common mechanism. In particular the more prevalent “class I” fusion proteins harbor two heptad repeat (HR) regions which are central to the process, the first one in the N-terminal region, adjacent to the fusion peptide (HRN), and the second one in the C-terminal region, immediately preceding the transmembrane domain (HRC). The currently accepted model stipulates that once fusion is initiated by the binding of the envelope glycoprotein (gp) to its cellular receptor, the HRN and HRC regions become separated in the so-called prehairpin intermediate, which bridges the viral and cell membranes; in the prehairpin structure, HRN forms a trimeric coiled-coil. Folding of the HRC onto the HRN trimer leads to the formation of a 6-helical bundle (6HB), and in this process the two membranes are driven in close apposition, ultimately resulting in their fusion, as reviewed in [1]. In this view, inhibitors that bind to the prehairpin intermediate and prevent its transition to the 6HB inhibit viral entry. This is the case for enfuvirtide, which spans part of the HRC region of the fusogenic protein gp41 of HIV-1 [2]. The same mechanism of inhibition applies to peptides derived from the HR regions of many other viruses [3], [4], [5], [6], [7], [8], [9], [10], [11], [12], [13], [14], [15], [16], [17], [18], [19].

The characteristic sequence pattern of the heptad repeats, which drive formation of coiled coils, provides an important advantage for the development of peptide-based antivirals: putative HRN and HRC peptides in viral fusion proteins can be easily identified directly from genomic information, through computer programs like LearnCoil [20] or MultiCoil [21], [22]. Indeed, for the emerging coronavirus that in recent years caused the SARS outbreak [23], identification of HRN and HRC regions [24] and development of peptide inhibitors [17], [19] *preceded* the determination of the structure of the fusion protein [25], [26], [27], [28].

Therefore, in addition to being a viable strategy for known viruses, inhibition of viral fusion offers the opportunity for a rapid response to emerging viral pathogens, opening the possibility to develop a *specific* antiviral in very short timeframe.

However, the potency of HR-derived peptides differs considerably among enveloped viruses, with HIV being a particularly favorable case. The reasons for this difference are not fully established, although a major factor is represented by fusion kinetics. Indeed, viruses with a slower transition through the hairpin intermediate offer a longer window of opportunity for peptide inhibitors that, accordingly, show greater potency [29], [30], [31].

Several peptide engineering strategies have been successfully applied to increase the potency of peptide fusion inhibitors [8], [32], [33], [34], [35], [36], [37]. However, they all require sequence-specific modifications, a relatively time-consuming optimization process. We have taken a different approach, seeking ways to increase antiviral activity without the need to change the native HR-derived sequence.

To this end, we have previously reported that by attaching a cholesterol group to a peptide fusion inhibitor (“cholesterol-tagging”) we can dramatically increase its antiviral potency [38]. Cholesterol-tagging of C34, a peptide from the HRC domain of HIV-1 gp41, produced an inhibitor (C34-chol) that, depending on the viral strain tested, is 25- to 100-fold more potent than unconjugated C34, and 50- to 400-fold more potent than the clinical drug enfuvirtide [38].

We showed that this is the result of selectively enriching the peptide in the membrane, where viral fusion occurs [39], [40], [41], [42]. Cholesterol-tagging therefore appears to be a general method, applicable to many enveloped viruses.

We have recently shown that cholesterol tagging of an HRC peptide from the paramyxovirus human parainfluenza virus type 3 (HPIV3) also increased its antiviral potency [43]. Notably, this peptide maintained the ability of the unconjugated peptide to inhibit both HPIV3 and the emerging paramyxoviruses Hendra virus (HeV) and Nipah virus (NiV) [15], [44], both fatal in humans. The cholesterol-tagged peptide now acquired the ability to also inhibit the rubulavirus SV5 (also known as PIV5) [43]. For Nipah virus, the cholesterol-tagged HRC peptide was active *in vivo*, effectively preventing and treating in an established animal model what would otherwise be fatal Nipah virus encephalitis [45].

An additional advantage of cholesterol tagging is that it prolongs the half-life of the peptide *in vivo*, through binding to serum proteins. A pharmacokinetic (PK) study of C34-Chol in mice at the concentration of 3.5 mg/kg showed that whereas unconjugated C34 was undetectable in plasma after 6 h, 130 nM of C34-Chol was still detectable 24 h after the injection: a concentration ≈ 300-fold higher than the IC_90_ measured against multiple HIV-1 strains [38]. To further expand on the generality of our method to potentiate the activity of natural HRC peptide inhibitors, we assessed the value of dimerization of the fusion inhibitor. Several examples of the successful use of multimerization to increase the potency of fusion inhibitors [46], [47] or entry inhibitors [48] have been reported.

In the hypothesized avidity effect [49], dimerization/multimerization works by reducing the k*_off_* of the inhibitor-fusion protein complex formation. Cholesterol tagging, on the other hand, facilitates the encounter of the peptide with the viral target protein by enriching the peptide in target membranes, an effect which increases the k*_on_* for complex formation. The two modifications could therefore work in concert, and both can be implemented without any requirement for optimization of the native sequence of the HRN/HRC peptide.

In the following, we report on our design of cholesterol-tagged dimeric fusion inhibitors, and demonstrate their efficacy in preventing or curtailing HPIV3, NiV, and HIV-1 replication *in vitro* and *in vivo*.

## Results

### Design and Synthesis of the Cholesterol-containing Branching Core

In designing a moiety containing the cholesterol group and the branching functionalities we incorporated the features listed below.

Firstly, we included a single cholesterol group. On one hand, given the considerable reduction of solubility from the cholesterol moiety, we wanted to avoid multiple copies of it; on the other hand, we hypothesized that the balance of cholesterol to the polypeptide moiety would still provide efficient anchoring of the multimeric inhibitor to membrane rafts. A single anchoring moiety is sufficient for proteins considerably larger in size [50] than even large copy number multimeric inhibitors with a monomer size of 35–40 amino acids. Data published after our work was completed lend further support to our hypothesis [51]. Moreover, we envisage having only two copies of the inhibitor sequence, as discussed below.

Secondly, we decided to focus on dimeric inhibitors. Findings by Kay and co-workers suggest that despite the trimeric structure of the fusion intermediate, the maximum benefit of multimerization is achieved at the level of dimer, with little – or even no – additional benefit in trimeric inhibitors [46], [47].

**Figure 1:**
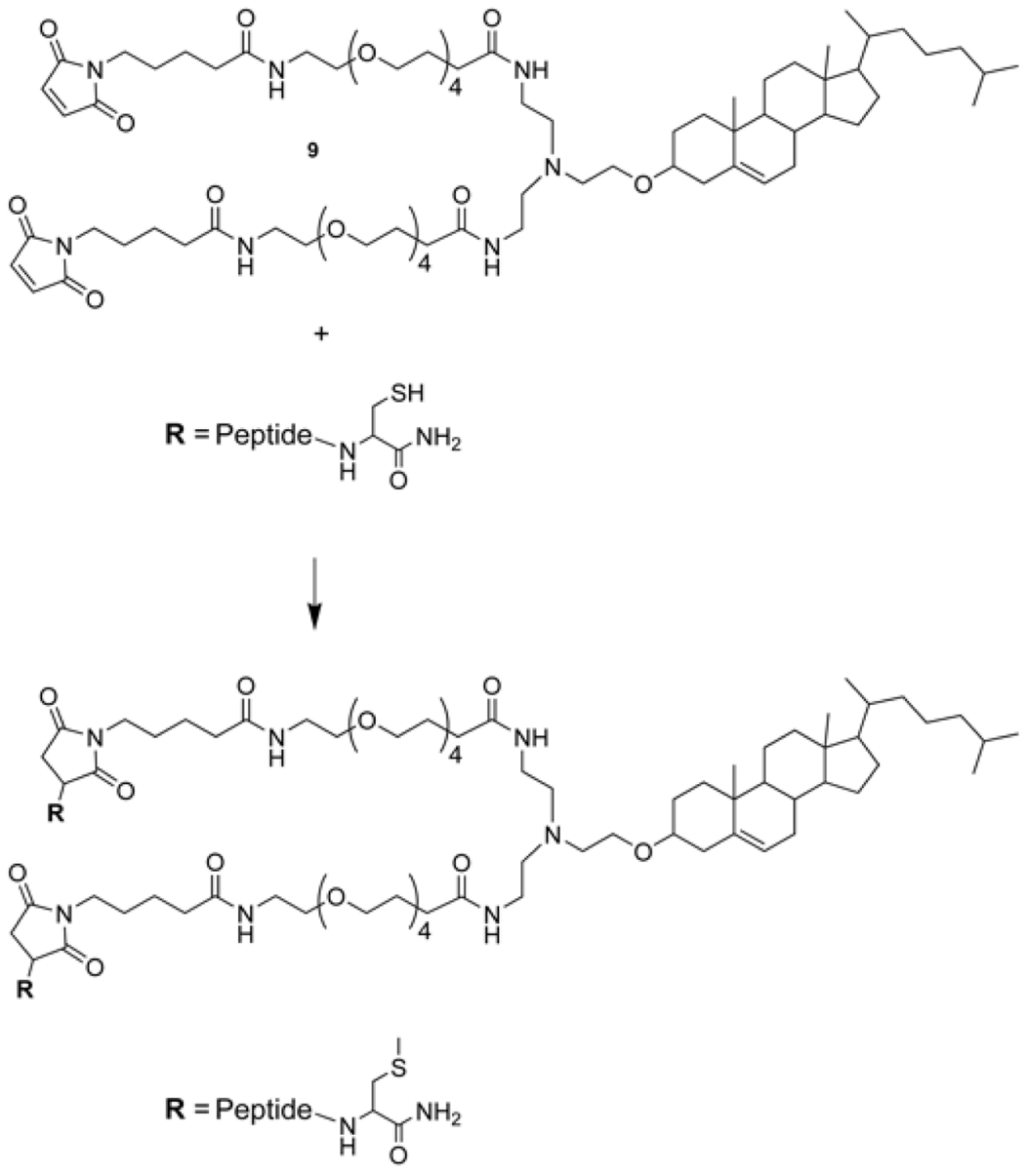
Synthesis of the dimeric cholesterol-tagged fusion inhibitors via conjugation of a cysteine-containing peptide precursor to the maleimide-functionalized core 9.

Thirdly, we selected four polyethylene glycol (PEG) units as the spacer between each monomer and the cholesterol core (Figure 1). This would provide approximately the same distance between monomers as that successfully used by Welch *et al*. [46]. Moreover, we had found a PEG_4_ spacer between the peptide and cholesterol to be beneficial in monomeric fusion inhibitors [45]. Notably, in a subsequent publication Welch et al. showed that reduction of the linker length provided considerable increase in potency [47]. This is clearly an area that requires further exploration in our next-generation branching cores.

Fourthly, we selected maleimide as a well-established chemoselective reactive moiety to conjugate the cholesterol-containing core to the cysteine-containing peptide.

The structure of the cholesterol-containing dimeric core **9** is shown in Figure 1. The compound is stable, and when stored at –20°C it can be used reliably for at least 12 months.

### Synthesis of Dimeric Cholesterol-tagged Fusion Inhibitors

The peptides used in this study are listed in Table 1. The dimeric cholesterol-tagged fusion inhibitors were prepared via conjugation of the cysteine-containing peptide precursors (HPIV-P1, HIV-P1) to the maleimide-functionalized core **9**. We had initially considered a bromoacetyl group as the reactive moiety, as we had done for the monomeric cholesterol-tagged inhibitors [38], [43], [45]. The corresponding functionalized core was prepared as described for **9** (see Figures S1 and S2), by using in the last step bromoacetyl anhydride instead of 4-maleimido-butyric acid. However, it turned out to be difficult to control the competition between reactivity of the core with the peptide and hydrolysis of the bromoacetyl group, leading to a difficult-to-separate mixture of dimeric and momomeric/capped conjugates. Using maleimide instead as the reactive moiety, the reaction was straightforward, and it proceeded rapidly in good overall yield.

**Table 1:** Sequence of the peptides used in this study.

Peptide	Sequence
HPIV-P1	Ac-VALDPIDISIVLNKAKSDLEESKEWIRRSNGKLDSI-GSGSG-C-NH_2_
HPIV-P2	Ac-VALDPIDISIVLNKAKSDLEESKEWIRRSNGKLDSI-GSGSG-C(CH_2_CONH_2_)-NH_2_
HPIV-P3	Ac-VALDPIDISIVLNKAKSDLEESKEWIRRSNGKLDSI-GSGSG-C(PEG_4_-Chol)-NH_2_
HPIV-P4	[(Ac-VALDPIDISIVLNKAKSDLEESKEWIRRSNGKLDSI-GSGSG-C(MAL-PEG_4_)]_2_-Chol
HPIV-P5	[(Ac-VALDPIDISIVLNKAKSDLEESKEWIRRSNGKLDSI-GSGSG-C(MAL-PEG_11_)]_2_-NH_2_
HIV-P1	Ac-WMEWDREINNYTSLIHSLIEESQNQQEKNEQELL-GSG-C-NH_2_
HIV-P2	Ac-WMEWDREINNYTSLIHSLIEESQNQQEKNEQELL-GSG-C(CH_2_CONH_2_)-NH_2_
HIV-P3	Ac-WMEWDREINNYTSLIHSLIEESQNQQEKNEQELL-GSG-C(PEG_4_-Chol)-NH_2_
HIV-P4	[(Ac-WMEWDREINNYTSLIHSLIEESQNQQEKNEQELL-GSG-C(MAL-PEG_4_)]_2_-Chol
HIV-P5	[(Ac-WMEWDREINNYTSLIHSLIEESQNQQEKNEQELL-GSG-C(MAL-PEG_11_)]_2_-NH_2_

### The Cholesterol-tagged Dimers Show Potent Antiviral Activity Against Different Enveloped Viruses

We initially focused on HPIV3 and NiV. These viruses represent major unmet medical needs [52], [53], and our previous finding that the HRC peptide derived from the sequence of HPIV3 is effective on both HPIV3 and NiV [15], [44] raised the possibility of developing a single inhibitor for both. Moreover, cholesterol-tagging of this peptide considerably improved its antiviral potency on both viruses [43]. However, the cholesterol-tagged inhibitor was not yet potent enough since it required sequence optimization to display activity *in vivo* [45]. This sequence therefore offered an ideal opportunity to test if dimerization coupled to cholesterol-tagging could provide further potency enhancement in comparison with cholesterol-tagging alone.

**Figure 2:**
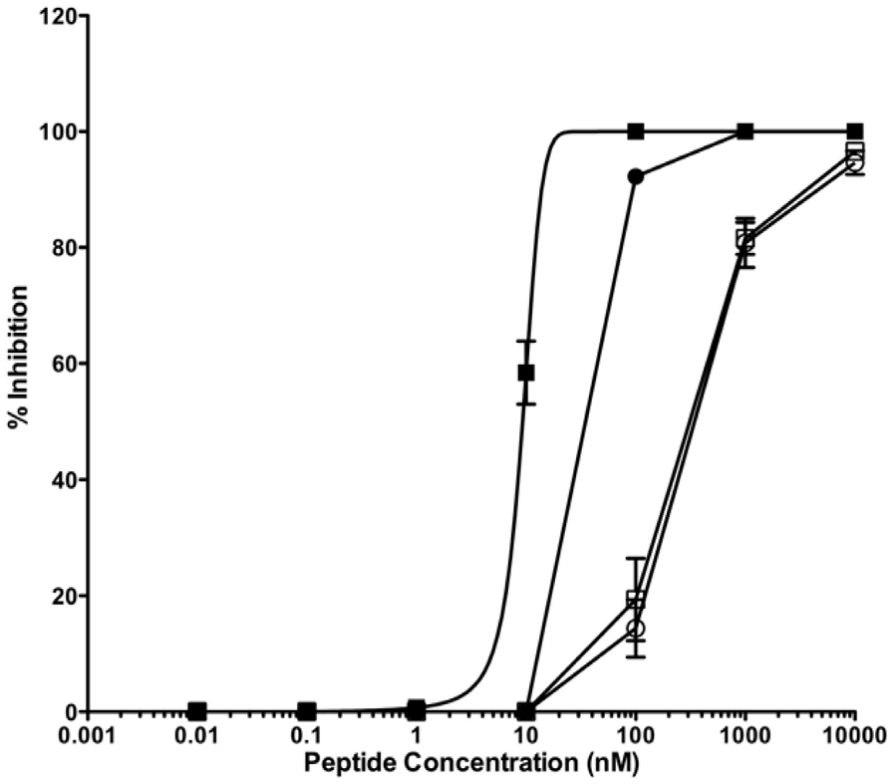
Inhibition of type 3 HPIV envelope glycoproteins mediated fusion by the HRC-derived fusion inhibitors HPIV-P2 (○), the monomeric cholesterol-tagged fusion inhibitor HPIV-P3 (•), the dimeric fusion inhibitor HPIV-P5 (□), and the dimeric cholesterol-tagged inhibitor HPIV-P4 (▪). The percent inhibition of fusion (compared to control cells not treated with peptide) is shown as a function of the (log-scale) concentration of peptide. The values are means (±SD) of the results from three experiments.

**Figure 3:**
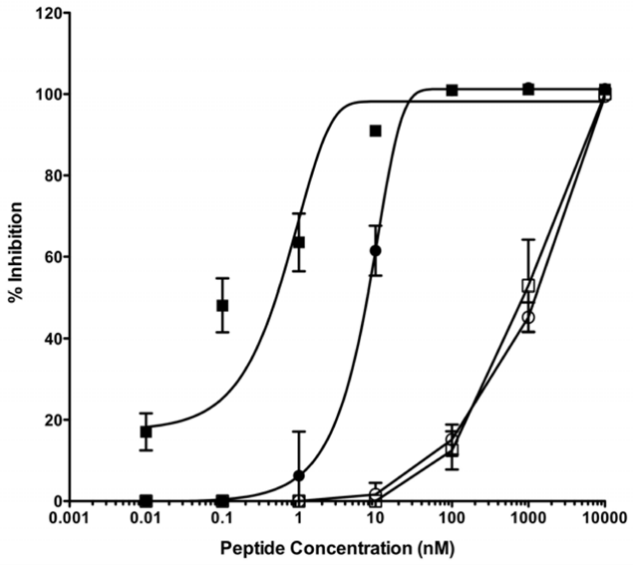
Inhibition of Nipah virus envelope glycoproteins mediated fusion by the HPIV-3 HRC-derived fusion inhibitors HPIV-P2 (○), the monomeric cholesterol-tagged fusion inhibitor HPIV-P3 (•), the dimeric fusion inhibitor HPIV-P5 (□), and the dimeric cholesterol-tagged inhibitor HPIV-P4 (▪). The percent inhibition of fusion (compared to control cells not treated with peptide) is shown as a function of the (log-scale) concentration of peptide. The values are means (±SD) of the results from three experiments.

We have recently shown that peptide efficacy in vivo is directly correlated with inhibition of viral fusion and cell-to-cell spreading. Peptide inhibitors able to inhibit viral entry but inefficient at blocking cell-to-cell fusion were not effective in the natural host [45], [54]. Based on these data we decided to use a cell-to-cell fusion assay using cells co-expressing the envelope viral glycoproteins of HPIV3 (Figure 2) and NiV (Figure 3) which is our most stringent in vitro assay to evaluate peptide efficacy Figure 2 shows a comparison of the fusion inhibitory activity for HPIV3, of the cholesterol-tagged monomer HPIV-P3 and the cholesterol-tagged dimer HPIV-P4, derived from the same peptide sequence; for comparison, we also show the fusion inhibitory activity of the native, untagged peptide inhibitor HPIV-P2, and of a control peptide dimerized via a PEG_11_ linker, HPIV-P5, similar to the one used by Welch *et al*. [46].

The cholesterol-tagged dimer is considerably more potent than the cholesterol-tagged monomer, which in turn is ≈ 10-fold more potent than the untagged inhibitor. Notably, in this case no advantage is observed for dimerization alone, which exerts a positive effect only in the presence of the cholesterol moiety.

Encouraged by these data, we tested the cholesterol-tagged dimer for NiV: the HPIV3-derived HRC peptide is *more* active on this virus [15], [44]. Figure 3 shows a comparison of the fusion inhibitory activity on NiV fusion of the monomeric fusion inhibitor HPIV-P2 [44], the monomeric cholesterol-tagged inhibitor HPIV-P3 [43], the dimeric inhibitor HPIV-P5, and the dimeric cholesterol-tagged inhibitor HPIV-P4. Also in this case, the dimeric-cholesterol tagged inhibitor is ≈ 10-fold more potent than the cholesterol-tagged monomer, while the untagged monomer and untagged dimer show similar (lower) potency.

In these experiments, there is progress from the native HRC sequence to more and more potent inhibitors, without the need for extensive sequence optimization. A relatively weak fusion inhibitor (IC_50_>100 nM) is turned into a reasonably potent peptide (IC_50_ between 10–100 nM for HPIV3, between 1–10 nM for NiV) and then into a potent inhibitor (IC_50_<10 nM for HPIV3, IC_50_<1 nM for NiV).

We confirmed the in vitro efficacy using NiV live virus in plaque reduction assays and we found that the IC_50_ values are 8.02±0.66 nM for the cholesterol tagged monomer and 4.36±1.3 nM for the cholesterol tagged dimer. As we have previously shown for HPIV3 [45] the cholesterol tagged peptides are highly effective at blocking viral entry and the increased potency due to dimerization could be appreciated in the cell-to-cell fusion assay of figure 2 and 3.

We then turned our attention to HIV-1. In this regard, we were facing the opposite end of the spectrum in terms of potency enhancement, since the native HRC peptide is already a potent fusion inhibitor, and the potency of the cholesterol-tagged peptide is in the picomolar range [38]. Moreover, access to the gp41 prehairpin intermediate appears to be sterically restricted, with limited access to large proteins [55], [56], [57], which would eliminate the potential beneficial effects of multimerization.

**Figure 4:**
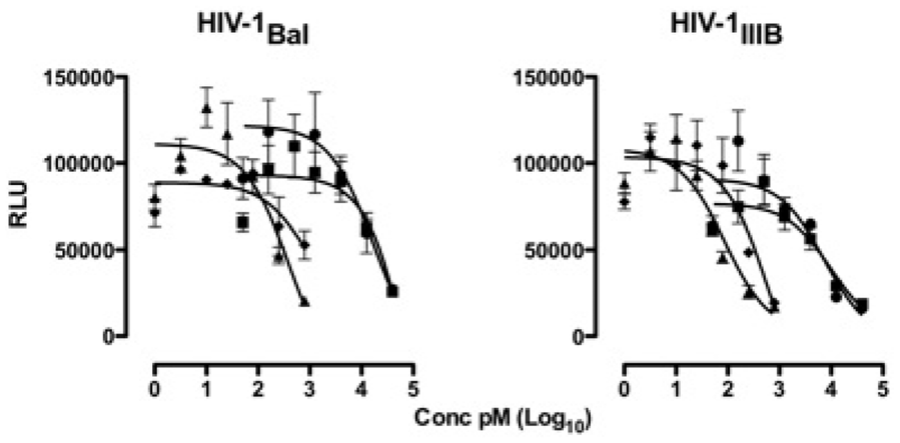
Inhibition of HIV infectivity by the fusion inhibitor HIV-P2 (▪), the monomeric cholesterol-tagged inhibitor HIV-P3 (p), the dimeric fusion inhibitor HIV-P5 (•), and the dimeric cholesterol-tagged inhibitor HIV-P4 (⧫). The efficiency of virus infection is shown as relative luciferase units (RLU) as a function of the (log-scale) concentration of peptide.

We compared the inhibitory activity on HIV-1 infectivity of the untagged fusion inhibitor HIV-P2, the monomeric cholesterol-tagged inhibitor HIV-P3 [38], the dimeric cholesterol-tagged inhibitor HIV-P4, and the corresponding dimer without the cholesterol tag, HIV-P5. Two strains of HIV-1 were used, the CCR5 coreceptor-tropic (R5) strain BaL and the CXCR4 coreptor-tropic (X4) strain LAI/IIIB, as shown in Figure 4.

The cholesterol-tagged dimer is an extremely potent inhibitor of both the IIIB and BaL strains, with IC_50_ = 210±370 pM and 10±10 pM, respectively; the potency increase versus the untagged HR-derived peptide is 150-fold and 6,000-fold on the two strains (IC_50_ for HIV-P2 = 31.12±36 nM and 60.14±84 nM on IIIB and BaL). The monomeric cholesterol-tagged inhibitor is comparably potent, with IC_50_ = 80±70 pM and 360±330 pM on IIIB and BaL.

The comparable potency of the cholesterol-tagged monomer and dimer suggest that we have reached the previously identified potency plateau for very high-affinity inhibitors, where an increase in the stability of the 6HB formed by the peptide does not translate into higher antiviral potency [32], [46], [47]. Kay and co-workers have shown that for inhibitors reaching beyond this threshold, the extra-affinity can represent a “resistance capacitor”, i.e. a reserve of binding energy that counteracts the onset of resistance due to affinity-disrupting mutations [46], [47]. We are currently investigating whether this is the case for the cholesterol-tagged HIV dimeric inhibitors.

### The Cholesterol-tagged Dimer is Efficacious *in vivo*

Based on these data, we selected the HPIV/NiV inhibitor for *in vivo* testing. We have previously shown that golden hamsters are a suitable small animal model for NiV infection [45], since they develop CNS manifestations of disease similar to the human disease, unlike the cat [58] or the ferret [59], which develop primarily respiratory disease.

**Figure 5:**
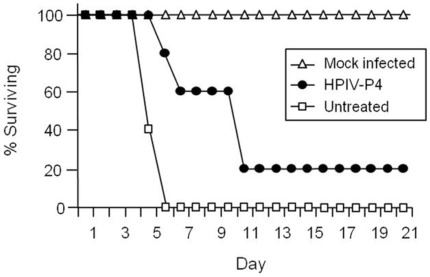
In vivo efficacy of the dimeric cholesterol-tagged peptide. HPIV-P4 (•) was given intraperitoneally to groups of 5 hamsters concurrently with live NiV infection. Injections of the inhibitor were then repeated every day for up to 10 days post infection. Control animals were injected with vehicle alone (untreated, □) or with peptide without NiV infection (mock infected, Δ). Treatment with the dimeric cholesterol tagged peptide led to a statistically significant increase of survival compared to non treated infected animals (Chi^2^ test = **P value = 0.008).

The efficacy of HPIV-P4 was investigated by administering the peptide to golden hamsters intraperitoneally concurrently with virus infection, at the dose of 2 mg/kg. Injections of the peptide were then repeated every day for up to 14 days post-infection. Hamsters were infected by intraperitoneal inoculation of NiV, using 500x the LD_50_. The infected hamsters were observed daily for clinical signs (prostration, neurological signs) and variations in temperature and weight. All the untreated animals were euthanized within 7 days after infection due to neurological disease (Figure 5). Treatment with the peptide resulted in survival of one of the five animals, while the onset of the illness was considerably delayed in the other four, leading to a significantly extended lifespan (Figure 5). This *in vivo* activity provides the proof of concept for anti-viral efficacy of dimerized, cholesterol-tagged peptides. Injection of the peptide in the absence of virus challenge did not result in any apparent toxicity, with all the animals in the mock-infected group being healthy at the end of the observation period (Figure 5).

## Discussion

In conclusion, we have shown that dimerization of an HRC-derived fusion inhibitory peptide in conjunction with cholesterol tagging may be a general strategy for increasing its antiviral potency *in vitro*, and that dimers can be effective *in vivo*. This observation complements our previous reports on cholesterol-tagging of fusion inhibitors [38], [43], [45], [60], [61].

We envisage several areas of improvement over the current inhibitor series. Optimization of the length of the linker joining the monomers may provide considerable increase in potency [49], as specifically demonstrated for HIV by the work of Kay and co-workers [47]. Using the synthetic scheme shown in Figures S1, S2, maleimide-functionalized cores with PEG spacers of different length can be easily prepared, and they can all be reacted with the same precursor to rapidly select the most potent inhibitor. Also, although we focused on dimeric peptides, trimeric or higher copy number cores may provide an advantage, especially when the starting HRC inhibitor is weak. Finally, other chemoselective pairs may prove superior in terms of reactivity and yield over the maleimide-cysteine pair. It should be noted however, that the latter pair offers a safety advantage, since it is featured in an antibody-drug conjugate recently approved for human use (ADCETRIS®, Seattle Genetics).

At this stage, cholesterol-tagging and dimerization represent feasible strategies for boosting the inhibitory potency to levels that, in some cases, may be compatible with *in vivo* use, without the need for extensive sequence optimization. With the availability of the necessary building blocks, synthesis of cholesterol-tagged monomers and dimers is easily and rapidly accomplished from the same precursor peptide. As such, this strategy may contribute to emergency responses to outbreaks of existing or novel viruses. The activity *in vivo* of a dimeric cholesterol-tagged inhibitor of NiV supports the potential utility of this strategy. We are currently exploring the scope of this approach on several enveloped viruses.

## Materials and Methods

### Synthesis

The synthesis of the cholesterol-containing branching core [Mal-PEG_4_]_2_-Chol (**9**) is detailed in the Figures S1 and S2. The synthesis of peptides HPIV-P1, HPIV-P2, HPIV-P3, HIV-P1, HIV-P2, and HIV-P3 (Table 1) has already been described [38], [43]. Chemoselective conjugation between the Cys-peptide precursors HPIV-P1 and HIV-P1 and the cholesterol derivative **9**, to yield conjugates HPIV-P4 and HIV-P4 (Table 1) is detailed in the Figures S1 and S2.

### Virus

Nipah virus (isolate UMMC1; GenBank AY029767) [62] was prepared on Vero-E6 cells as described previously [63] in biosafety level 4 (BSL-4) laboratory Jean Merieux in Lyon, France.

### Antiviral Assays

The assays to measure Peptide inhibition of viral entry or viral infectivity were performed as previously described [38], [43], [45]. A brief description of the assays is given below.

#### Antiviral activity against live Nipah virus

In the plaque reduction assay, serial dilutions of peptides in Dulbecco’s modified Eagle’s medium (DMEM) (Invitrogen) were added to reporter cell monolayers of Vero E6 cells (in 12well-plates, 5.10^5^ cells/well) for 1h at 37°C, 5% CO_2_. Then, 200 pfus of NiV were added to the supernatant of the Vero cells and incubated for 1h at 37°C. Supernatants were then replaced by 2ml of 1.6% of Carboxymethylcellulose in DMEM containing 3% of FCS. Plates were incubated for 4 days at 37°C, 5% CO_2_. Plates were finally revealed as a classic central plaque assay, as previously described in Guillaume et al 2004 (REF). IC_50_ were calculated using Graph Pad Prism4 software. Data were expressed as mean±standard deviation (SD).

#### Antiviral activity against HIV-1 in TZM-bl cells

The TZM-bl cell line is an epithelial HeLa-derived cell line engineered to express CD4, CCR5, CXCR4 and integrated reporter genes for firefly luciferase (Luc) and *E.coli* β-gal under the control of an HIV-1 LTR that is transactivated by the Tat protein synthesized after infection [4]. Cells were seeded into 96-well flat bottomed plastic plates (Falcon, Becton Dickinson Labware, Lincoln Park, NJ) at 1×10^4^ cells/well and were incubated in the presence or absence of the compounds at 37°C for 1 h in a total volume of 50 µl. Aliquots of CCR5-dependent (R5) HIV-1_Bal_ or CXCR4-dependent (X4) HIV-1_LAI/IIIB_ viral stocks at the multiplicity of infection (moi) of 4 were then added to a total volume of 100 µl in culture medium containing 15 µg/ml DEAE-dextran (Sigma-Aldrich, St. Louis, MO). Forty-eight h later, the efficiency of virus infection was determined as relative Luc units (RLU) by Luciferase assay system (Promega, Madison, WI).

#### Antiviral activity against HIV-1 in activated peripheral blood mononuclear cells (PBMC)

PBMC were isolated from blood obtained at the New York Blood Center, then cultured in RPMI 1640 supplemented with 10% FBS, 10 mM L-glutamine (Cellgro), and 100 U/ml IL-2 (ARRRP, contributed by Hoffman-LaRoche, Inc - Culture Medium). Half the PBMC culture was stimulated with 5 µg/ml of phytohemagglutinin (PHA; Sigma, St. Louis, MO), half with an anti-CD3 mAb (supernatant from the OKT3 hybridoma) in Culture Medium, directly after isolation. After 3 days, the differently stimulated cells were mixed in equal proportions, washed, and suspended in Culture Medium supplemented with 100 µg/ml of penicillin/streptomycin for the remainder of the culture. The PBMC were seeded at 1.4×10^6^ cells/ml in 100 µl per well in 96-well plates before addition of a serially diluted inhibitor immediately prior to inoculation with the test virus. HIV-1 replication was measured using an in-house p24 Gag antigen ELISA after 7 days of culture. Residual p24 Gag from the input virus in 4 replicates per assay was measured and subtracted. Net p24 Gag production in the test wells was compared with that in the control wells (no inhibitor in 12 replicates/assay, defined as 100%). The inhibitors were titrated in 5-fold steps and each culture well, mixed cells and supernatant, was analyzed. Two assay replicates per condition were combined in the ELISA, e.g. 12 assay 100% viral growth control wells (see above) were combined to 6 ELISA wells.

### Beta-galactosidase Complementation-based Fusion Assay

We adapted an assay [64] that detects early stages of fusion activation, since the read-out does not depend upon downstream transactivation events. We used this assay for experiments in which the greater range of detection of this assay was necessary. The assay is based on alpha complementation of beta-gal; the beta-gal protein lacking the N-terminal 85 residues (omega peptide) is expressed from one plasmid, and the N-terminal 85 residues (alpha peptide) is expressed from a second plasmid. Cell fusion leads to complementation, and beta-galactosidase is quantitated using the Galacto-Star (Applied Biosystems) chemiluminescent reporter gene assay system. Receptor bearing cells expressing the omega peptide are mixed with viral glycoproteins co-expressing cells that also express the alpha peptide, at various temperatures and at specific time points. Fusion is stopped by lysing the cells with lysis buffer. Fusion inhibitory peptides are added when the two set of cells are mixed together.

### Golden Hamster Infection

Eight-week-old golden hamsters (*Mesocricetus auratus*, Janvier, France) were anesthetized and infected intraperitoneally (i.p.) with 0.4 mL of NiV in a BSL-4 laboratory. Groups of 5 animals were treated daily by i.p injections of peptide (2 mg/kg) for 10 days, starting from the day of infection, or left untreated. The animals were followed daily for 3 weeks. For comparison of survival curves, Chi^2^ analysis was performed using test using Graph Pad Prism4 program.

### Ethics Statement

All animals were handled in strict accordance with good animal practice as defined by the French national charter on the ethics of animal experimentation and all efforts were made to minimize suffering. Animal work was approved by the Regional ethical committee (Comité Ethique pour l’Experimentation Animal, CECCAPP, protocol P4_2010_008) and experiments were performed in the INSERM Jean Mérieux BSL-4 laboratory in Lyon, France (French Animal regulation committee N° B69 387 05 02).

## Supporting Information

Figure S1 **Synthetic scheme for the cholesterol-containing branching core 5.**
(DOC)

Figure S2 **Synthetic scheme for the maleimide-functionalized cholesterol-containing branching core 9.**
(DOC)
