# Supplementary material for: A General Strategy to Endow Natural Fusion-protein-Derived Peptides with Potent Antiviral Activity
Source: PLoS One. 2012 May 16;7(5):e36833. doi: 10.1371/journal.pone.0036833 (PMC3353973; doi:10.1371/journal.pone.0036833)
Supplement: Figure S2 — Synthetic scheme for the maleimide-functionalized cholesterol-containing branching core 9. (DOC) [file pone.0036833.s002.doc]

Supplemental Material for

**A General Strategy to Endow Natural Fusion-protein-Derived Peptides with Potent Antiviral Activity In Vivo.**

Antonello Pessi^a^*, Annunziata Langella^b^, Elena Capitò^c^, Silvia Ghezzi^d^, Elisa Vicenzi^d^, Guido Poli^e,f^, Thomas Ketas^g^, Cyrille Mathieu^i,1^, Riccardo Cortese^h^, Branka Horvat^i^, Anne Moscona^g,l^, and Matteo Porotto^l^*.

**Synthesis of [Mal-PEG_4_]_2_-Chol (9)**

**Synthesis of 1 (Figure S1).** To a flask containing bis(2-aminoethyl)amine (1 g, 9.7 mmol) in 50 mL of THF, cooled at 0 ºC, was added Et_3_N (4.06 mL, 29.1 mmol), and then dropwise 2-(tert-Butoxycarbonyloxyimino)-2-phenylacetonitrile. The mixture was stirred for 1h at 0 ºC and then 2h at room temperature. After evaporation of the solvent *in vacuo*, the residue was dissolved in CH_2_Cl_2_, washed with 1N NaOH, dried over Na_2_SO_4_, filtered and concentrated, to obtain 2.76 g of **1** as a yellow oil (yield, 94%).

**Synthesis of 2 (Figure S1).** Cholesterol (1 g, 2.6 mmol) was dissolved in 40 mL of THF/DMF (1:1) and 60% sodium hydride (w/w) in mineral oil (0.6 g, 15.5 mmol) was added, followed by stirring for 10 min. 2-bromo-1,1-dimethoxyethane (1.21 mL, 7.8 mmol) was added dropwise, and the mixture was stirred at 90 ºC under reflux for 18 h. The mixture was cooled and CH_2_Cl_2_/MeOH (1:1) was added to eliminate excess NaH. After elimination of solvent *in vacuo*, the residue was taken up in EtOAc, washed several times with water, dried over Na_2_SO_4_, filtered and concentrated. The crude was purified by flash column chromatography (BIOTAGE) on silica gel using a gradient of 2-10% petrol ether in EtOAc, to obtain 1.23 g of **2** as a white solid (yield, 94%).

**Synthesis of 3 (Figure S1).** Trifluoroacetic acid (TFA)/water (1:1) (2.5 mL, 16.2 mmol) was added to a solution of **2** (0.5 g, 1 mmol) in 10 mL of CH_2_Cl_2_, and the mixture was stirred at room temperature for 6h. The mixture was neutralized with 1N NaOH, extracted twice with CH_2_Cl_2_, dried over Na_2_SO_4_, filtered and concentrated, to obtain **3** as a white solid, that was used directly in the next step.

**Synthesis of 4 (Figure S1).** **3** (1.47 mmol) was dissolved in 40 mL of MeOH containing **1** (0.535 g, 1.77 mmol), and Et_3_N (0.611 mL, 4.41 mmol), AcOH (0.42 mL, 7.35 mmol), and NaBH_3_CN (0.185 g, 2.94 mmol) were added in succession. The mixture was stirred for 18 h at room temperature, diluted with EtOAc, washed twice with NaHCO_3_ and water, dried over Na_2_SO_4_, filtered and concentrated. The crude was purified by flash column chromatography (BIOTAGE) on silica gel using a gradient of 0-8% MeOH in CH_2_Cl_2_, to obtain 0.95 g of **4** as a white solid (yield, 90%).

**Synthesis of 5 (Figure S2).** TFA (3.6 mL, 46.7 mmol) was added to a solution of **4** (1.12 g, 1.57 mmol) in 20 mL of CH_2_Cl_2_, and the mixture was stirred at room temperature for 4h. All the volatiles were removed *in vacuo* and the crude was lyophilized to obtain **6** as a brown solid, which was used directly in the next step.

**Synthesis of 7 (Figure S2)****.** Boc-amido-dPEG_4_™ acid (**6**) (1.15g, 3.14 mmol, commercial from Quanta BioDesign) was dissolved in 47 mL of CH_2_Cl_2_ together with O-Benzotriazole-N,N,N’,N’-tetramethyl-uronium-hexafluoro-phosphate (HBTU, 1.31 g, 3.45 mmol) and N,N'-diisopropylethylamine (DIPEA, 1.1 mL, 6.28 mmol). The mixture was stirred for 30 min at room temperature until complete dissolution of HBTU. **5** was added and the mixture stirred for 2h at room temperature. Since some mono-derivatized compound was still present, a new addition was made of **6** (365 mg, 1 mmol), HBTU (402 mg, 1.1 mmol), and DIPEA (0.350 mL, 2 mmol) dissolved in 30 mL of CH_2_Cl_2_. The mixture was stirred at room temperature for another hour, when the reaction was complete. After addition of water and CH_2_Cl_2_, the organic phase was separated, the aqueous phase extracted with CH_2_Cl_2_, and the combined organic phase was dried over Na_2_SO_4_, filtered and concentrated. The crude was purified by flash column chromatography (BIOTAGE) on silica gel using as solvent CH_2_Cl_2_/MeOH/Et_3_N (97.5:2:0.5) to obtain 1.88 g of **7** as a yellow oil (yield, 98%).

**Synthesis of 8 (Figure S2).** TFA (3.6 mL, 46.7 mmol) was added to a solution of **7** (1.88 g, 1.55 mmol) in 30 mL of CH_2_Cl_2_, and the mixture was stirred at room temperature for 3h. All the volatiles were removed *in vacuo* and the crude was lyophilized to obtain **8** as a brown oil, that was used directly in the next step.

**Synthesis of 9 (Figure S2).** 4-maleimido-butyric acid (0.284 g, 1.55 mmol) was dissolved in 25 mL of CH_2_Cl_2_ together with HBTU (0.617 g, 1.62 mmol) and DIPEA (0.57 mL, 3.26 mmol). 0.5 mL of DMF was added to help complete dissolution. **8** dissolved in 20 mL of CH_2_CL_2_ was added, and the mixture was stirred for 2h at room temperature, after which the reaction was complete. After addition of water and CH_2_Cl_2_, the organic phase was separated, the aqueous phase extracted with CH_2_Cl_2_, and the combined organic phase was dried over Na_2_SO_4_, filtered and concentrated. The crude was purified by flash column chromatography (BIOTAGE) on silica gel using a gradient of 4-15% MeOH in CH_2_Cl_2_, yielding 0.462 g of **9** as a white solid (yield, 56%).

**Peptide Conjugation to the cholesterol core 9.**

Chemoselective conjugation between the Cys-peptide precursors HPIV-P1 and HIV-P1 and the cholesterol derivative **9** (see Figure 1 in the manuscript), to yield conjugates HPIV-P4 and HIV-P4 (Table 1 in the manuscript) was carried out as follows:

**HPIV-P4**. 5 mg (3.7 µmol) of **9** dissolved in 0.4 mL of THF (10 mg/mL), were added to 30 mg of peptide HPIV-P1 (6.6 µmol) dissolved in 1.5 mL of DMSO (conc. 20 mg/mL). The reaction was monitored by UPLC-mass spectrometry on a SQ Detector Waters Platform with a Waters Acquity UPLC BEH300 C4 column (2.1 x 100 mm, 1.7 µm,) using as eluents (A) 0.1% TFA in water and (B) 0.1% TFA in acetonitrile, and the following linear gradient: 30%-30%(B) in 1 min, then 30%‒95%(B) in 4 min, flow 0.4 ml/min, T = 45 °C, λ = 214 nm; t_r_ = 3.02’. After 60 min the reaction was complete and it was quenched with glacial acetic acid to pH = 4, then water was added drop-wise up to the highest amount that did not induce precipitation.

*Purification and analytical characterization*. The resulting conjugate HPIV-P4 was purified by reverse-phase HPLC on a C_4_ DELTAPAK Waters cartridge, 300Å 20x100 mm, 15 µm; Flow: 80 mL/min: Gradient: 30%B isocratic (5 min) then linear to 70% B in 20 min;. Eluents: (A) 0.2% AcOH in water and (B) 0.2% AcOH in acetonitrile. AcOH was used to obtain the final compound as an acetate salt. Pooling of the cleanest fractions gave 15.4 mg of conjugate at ≥ 95% purity (yield: 51%). MW _theor_: 10426 Da, MW _found_: 10421 Da.

**HIV-P4**. 2.7 mg (2 µmol) of **9** dissolved in 0.3 mL of THF (10 mg/mL), were added to 20 mg of peptide HIV-P1 (4.3 µmol) dissolved in 1 mL of DMSO (conc. 20 mg/mL), followed by addition of 12.7 µL of DIPEA and 64 µL of a 70mM acqueous EDTA solution (pH =8). The reaction was monitored by UPLC-mass spectrometry on a SQ Detector Waters Platform with a Waters Acquity UPLC BEH300 C4 column (2.1 x 100 mm, 1.7 µm,) using as eluents (A) 0.1% TFA in water and (B) 0.1% TFA in acetonitrile, and the following linear gradient: 30%-30%(B) in 1 min, then– 30%-95%(B) in 4 min, flow 0.4 ml/min, T = 45 °C, λ = 214 nm; t_r_ = 3.02 min. After 30 min the reaction was complete and it was quenched with TFA to pH = 3, then water was added drop-wise up to the highest amount that did not induce precipitation.

*Purification and analytical characterization*. The resulting conjugate HIV-P4 was purified by reverse-phase HPLC on a C4 DELTAPAK Waters cartridge, 300Å 25x100 mm, 15 µm; Flow: 80 mL/min: Gradient: 30%B isocratic 5 min then linear to 70% B in 20 min; Eluents: (A) 0.2% TFA in water and (B) 0.2% TFA in acetonitrile. Pooling of the cleanest fractions gave 7.2 mg of (17) at ≥ 95% purity (yield: 35%) (MW: cal., 10528 Da, found, 10525 Da).

**Control peptide dimers without cholesterol tag**

The control peptide dimers HPIV-P5 and HIV-P5, lacking the cholesterol moiety, was prepared similarly, by reaction of the precursors HPIV-P1 and HIV-P1 with bis-Mal-dPEG_11_ (commercial from QuantaBiodesign). HPIV-P5: MW found: 9930, MW calc: 9931 Da; HPLC purity: 95%; yield: 42%; HIV-P5: MW found: 10031, MW calc: 10033 Da; HPLC purity: 96%; yield: 38%.

**Figure S2.** Synthetic scheme for the maleimide-functionalized cholesterol-containing branching core **9.**
